# The expenditure of computer-related worktime using clinical decision support systems in chronic pain therapy

**DOI:** 10.1186/s12871-015-0094-9

**Published:** 2015-08-01

**Authors:** Timm Hecht, Anika C. Bundscherer, Christoph L. Lassen, Nicole Lindenberg, Bernhard M. Graf, Karl-Peter Ittner, Christoph H. R. Wiese

**Affiliations:** Department of Anesthesiology, University Medical Centre Regensburg, Franz-Josef-Strauß-Allee 11, D-93053 Regensburg, Germany

**Keywords:** Drug-drug interaction, Clinical decision support systems (CDSS), Adverse drug reaction, Expenditure of time, Chronic pain, Polypharmacy

## Abstract

**Background:**

Estimate the expenditure of computer-related worktime resulting from the use of clinical decision support systems (CDSS) to prevent adverse drug reactions (ADR) among patients undergoing chronic pain therapy and compare the employed check systems with respect to performance and practicability.

**Methods:**

Data were collected retrospectively from 113 medical records of patients under chronic pain therapy during 2012/2013. Patient-specific medications were checked for potential drug-drug interactions (DDI) using two publicly available CDSS, Apotheken Umschau (AU) and Medscape (MS), and a commercially available CDSS AiD*Klinik*® (AID). The time needed to analyze patient pharmacotherapy for DDIs was taken with a stopwatch. Measurements included the time needed for running the analysis and printing the results. CDSS were compared with respect to the expenditure of time and usability. Only patient pharmacotherapies with at least two prescribed drugs and fitting the criteria of the corresponding CDSS were analyzed. Additionally, a qualitative evaluation of the used check systems was performed, employing a questionnaire asking five pain physicians to compare and rate the performance and practicability of the three CDSSs.

**Results:**

The AU tool took a total of 3:55:45 h with an average of 0:02:32 h for 93 analyzed patient regimens and led to the discovery of 261 DDIs. Using the Medscape interaction checker required a total of 1:28:35 h for 38 patients with an average of 0:01:58 h and a yield of 178 interactions. The CDSS AID required a total of 3:12:27 h for 97 patients with an average time of analysis of 0:01:59 h and the discovery of 170 DDIs. According to the pain physicians the CDSS AID was chosen as the preferred tool.

**Conclusions:**

Applying a CDSS to examine a patients drug regimen for potential DDIs causes an average extra expenditure of work time of 2:09 min, which extends patient treatment time by 25 % on average. Nevertheless, the authors believe that the extra expenditure of time employing a CDSS is outweighed by their benefits, including reduced ADR risks and safer clinical drug management.

## Background

Pharmacotherapy features prominently in patient treatment and the number of prescribed drugs has steadily increased [1]. According to literature estimates the average number is five prescribed drugs among the elderly patients [2, 3]. However, the growing number of drugs taken by patients is significantly associated with the risk of drug-drug interactions (DDIs) [3–5]. These DDIs may lead to adverse drug reactions (ADR) [6–8]. For example, each year up to 20.000 people die from ADRs in Great Britain’s hospitals [9], including ADRs due to DDIs. DDIs are not only an issue of polymedication but can also arise from lack of knowledge resulting in medication errors [10]. In fact, the majority of medication errors happens at the stage of prescription [11–14] making DDIs a rather common concern [12].

ADRs are a serious cause of mortality, morbidity and costs in the healthcare system, making them a major burden in healthcare [15–17]. Notably, there are estimates suggesting that up to 52 % of the ADRs are avoidable [18]. Thus, strategies to prevent ADRs are of special interest. Clinical decision support systems (CDSS) are especially conceived for this task [19, 20] and could positively affect physicians’ efforts concerning safe drug prescribing and the detection of DDIs [19–24]. However, not many data are available about how practicable it is to implement the usage of CDSSs in a hospital workflow. A major aspect for the decision to integrate the regular use of a CDSS in a clinical routine is the required time [11, 25–27]. Therefore, this study has compared three different CDSSs with respect to the expenditure of time, performance and practicability associated with the computer-based analysis of pharmacotherapies in order to prevent ADRs due to DDIs.

## Methods

### Study group

This was a retrospective clinical study in the chronic pain unit at the university hospital of Regensburg in Germany. The study population consisted of 113 patients who were undergoing chronic pain therapy at the chronic pain unit in the years 2012/2013. This setting was chosen because of the special interest in ADR resulting from DDIs in multi-morbid and poly-medicated patients. Patient records were viewed, extracting parameters such as age, body size, gender and weight for descriptive statistics. Furthermore, pain location and the duration of disease were recorded. Information about the patient’s medication was gathered, including the type and the total amount of drugs that had been prescribed.

### Check systems

For analyzing each patient’s pharmacotherapy for DDIs, three CDSS were selected: the two freely available internet-based interaction check systems: (1) “Apotheken Umschau” (AU; SCHOLZ Datenbank®, © ePrax AG) and (2) Medscape (MS; copyright © by WebMD LLC), and the commercially available (3) CDSS AiD*Klinik*® (AID; copyright © Dosing GmbH). Other CDSS are available in Germany. However, the AU, MS and AID check systems were chosen as they are representatives of the major types of CDSS: freely or commercially available, designed for use by both patients and physicians or by heath care professionals only, applicable to drugs approved in a specific country or internationally.

1. AU: The AU CDSS was designed for use by both patients and physicians. Therefore, the information provided is more detailed and comprehensible also for users without specific medical knowledge. The AU CDSS was conceived for use in Germany and drugs approved in Germany only. It is a tool providing the users not only with information about DDIs of a specific pharmacotherapy under consideration but additionally about double medication, fitness to drive, drug-food interactions and cumulative side effects. Up to ten drugs can be entered and analyzed using both, active agent and trade name, dose and dosage form. This CDSS classifies occurring DDI as: (1) Severe interactions that should be avoided implicitly. These are marked by the system with a red stop-sign. (2) Severe or significant interactions, that should be avoided, marked with a red exclamation mark. (3) Interactions that are significant but only in some rare cases, marked with orange exclamation mark. (4) Interactions of notable but minor relevance, marked with a yellow exclamation mark. (5) Interactions which most probably are not existent. Additionally, information about DDI is given, comprising a summary explaining the main interaction issue. Furthermore, albeit not considered in this study, the interaction is outlined in detail, classifying the interaction, reporting about the mechanism, time-dependent course, symptoms that can appear and which dose needs to be considered. Management of the interaction is recommended explaining which afflictions and symptoms are to be looked out for, which laboratory findings are important and which actions are to be taken. The CDSS AU drug databank is based on information approved by the Federal Institute for Drugs and Medical Devices and is continuously updated albeit at irregular intervals.
2. MS: Medscape (MS) is a freely available internet-based CDSS. It is an internationally well established tool for simple drug-drug interaction checking. It was created for English speaking countries and drugs approved in the USA only. Drugs are entered by generic name, active agent or trade name. A dose cannot be entered. The maximum number of drugs that can be analyzed is 30. A given patient’s pharmacotherapy is evaluated for DDIs. A short explanation about interaction issue and mechanism is provided. Furthermore, the tool indicates relevant aspects and risks of the patient’s regimen such as deterioration of renal function or renal dysfunction, the potential for causing gastrointestinal bleedings or teratogenic effects for certain drugs given during pregnancy. The CDSS classifies the DDIs as: (1) Serious - use alternative. (2) Significant - monitor closely. (3) Minor or non-significant. Of note, a DDI can be classified as serious, significant or minor at the same time when the underlying drug combinations have multiple, different effects. The clinical information is updated regularly and represents the expertise and practical knowledge of top physicians and pharmacists from leading academic medical centers in the United States and worldwide.
3. AID: AID, the third evaluated CDSS, is commercially available and was designed to support healthcare professionals in a hospital workflow. Drugs are entered by their generic name, active agent, trade name and dose. More than 30 drugs can be analyzed. Besides DDIs, users are informed about double medication, incompatibility, exceeded maximal doses and the risk of kidney insufficiency due to the patient’s pharmacotherapy. Furthermore, assistance is provided in drug usage during pregnancy. Interactions found are classified as (1) Clinically severe interaction, outlined red. (2) Clinically relevant interaction with potential to harm. (3) Clinically most probably not relevant interactions, outlined yellow. In addition, interactions are explained, pointing out risk patients and potential symptoms. Detailed clinical management is provided including the suggestion of alternative drugs, lowering or raising a drug dose or close monitoring if drug combinations avoided. The CDSS AID refers to the Pharmindex database which is updated every 14 days and issued by the Medizinischen Medien Informations GmbH (MMI, Neu-Isenburg, Germany).

### Pain physician questionnaire

Additionally, the three employed CDSSs were evaluated and compared by five pain physicians at the University of Regensburg to highlight the main advantages and weaknesses of each system. Furthermore, these physicians were asked to rate main advantages of each System to determine their preferred CDSS.

### Time measurement

To evaluate the expenditure of time using the CDSSs for analyzing patient’s pharmacotherapy time was taken with a stopwatch. For AU and MS time measurement involved: opening the Mozilla Firefox™ (Mozilla Foundation, Mountain View, California, United States) internet browser, selecting the CDSS, entering the patient’s medication, analyzing and printing the results. Printing the results in MS was eased by a print button whereas printing in AU was solely possible by text marking. Prior to their use, bookmarks for the websites of the MS and AU interaction checkers were attached to the bookmark library of the internet browser for faster access. This was neither required nor possible for AID.

For AID, the measured process contained: starting the hospital’s intranet, opening the AID homepage, choosing the “medi-box” item, entering the drugs, analyzing and printing the results using the print option. All interactions found were claimed DDI whether severe or non-significant and irrespective of whether or not the patient had experienced an ADR. This setup was chosen to simulate the drug interaction check as practiced under a normal work routine. Only patient’s regimens with at least 2 drugs and fitting the criteria of the corresponding CDSS were analyzed for DDIs.

### Definitions of the terms used

Drug-Drug-Interactions (DDI) were defined as reactions occurring from drugs taken at the same time and affecting each other. These reactions can be synergistic or antagonistic and influence the intended therapeutic effect as well as increase the possibility or the extent of side effects. The major issue of DDI is the potential to harm the patient.

Adverse drug events (ADE) comprise all adverse effects caused by normal as well as improper drug use. Adverse drug reactions (ADR) are specific ADEs. ADRs were defined as negative effects caused by the combination of two or more drugs, during regular use at normal dose, with the potential to harm a patient.

Medication error was defined as every drug combination leading to a DDI with potential to harm, even if no actual harm was done, the DDI was non-significant or the probability of causing harm was very small.

### Statistical analysis and ethics

Data were acquired primarily through the German Pain Questionnaire. Patient-specific parameters were encoded by a custom made system and entered in a table created with the spreadsheet program MS Excel, vs. 2010 (Microsoft Inc., Redmond, USA). For statistical analysis, data were processed by the statistics program IBM SPSS for Windows, vs. 20.0 (IBM SPSS Inc., New York, USA). To analyze statistical significance one-way ANOVA was performed. Descriptive data are presented as absolute values or percentages as indicated and partly expressed as the mean. The study was approved by the Local Ethics Commission (14-160-0069, Regensburg, Germany). According to the declaration of Helsinki, data were anonymized.

## Results

To investigate the time expenditure required to check for DDIs by the use of CDSS and compare their performance three different CDSS and a cohort of 113 chronic pain patients were employed. Additionally five pain physicians evaluated each CDSS to highlight advantages and weaknesses of the different check systems. Furthermore, pain physicians evaluations were compared to determine the preferred and most practical CDSS. Because of the purpose of the study, neither the clinical significance, nor the relevance or the correctness of the discovered interactions were considered.

### Chronic pain patients

113 records of patients undergoing pain therapy were analyzed. Descriptive statistics are shown in Table 1.

**Table 1:** Descriptive statistics

Gender^a^		Age (years)		Mean body weight (kg)		Patients height (cm)(mean)	
male	female	(Mean)	(Range)	80.6		170.4	
61 (54)	52 (46)	52.4	19 to 84				
Pain complaints for^a^							
>5 years	2-5 years	1-2 years	½-1 years	1 week-1 year		no declaration	
46 (40.7)	34 (30.1)	17 (15)	7 (6.2)	7 (6.2)		2 (1.8)	
Pain localization^a^							
Head	Cervical sp.	Thoracic sp.	Lumbar sp.	Upper limbs	Lower limbs	Abdominal	Whole-body
16 (14.2)	9 (8)	2 (1.8)	24 (21.2)	13 (11.5)	17 (15)	4 (3.5)	28 (24.8)
Maximum pain intensity^a^							
0-4	5-7			8-10		no declaration	
3 (2.7)	29 (25.7)			79 (69.9)		2 (1.7)	
Mean pain intensity^a^							
0-4	5-7		8-10		no declaration	Median	
16 (14.2)	47 (41.6)		47 (41.6)		3 (2.65)	7	

*sp* spine

^a^ Total number (percentage)

### Medication

Five hundred five drugs were prescribed to 110 patients; three of the former 113 patients had no medication at all. The minimum number of drugs among the patients with medication was one prescribed drug, the maximum number was twenty. The average number of drugs per patient was 4.59. 142 different types of drugs were prescribed. Most frequently prescribed drugs were analgesics (35.4 %). The non-opioid analgesic drugs with the highest frequencies of prescriptions were Ibuprofen (31.7 %), Metamizol (19.5 %) and acetylsalicylic acid (ASS) (17.8 %). Tilidin/Naloxone (30.9 %) and Tramadol (27.2 %) were the opioid-analgesics with the largest number of prescriptions. Drugs affecting the cardiovascular system (beta blockers, diuretics, statins, Ca^2+^-inhibitors, Angiotensin converting enzyme inhibitors (ACE-Inhibitors), Angiotensin-receptor-inhibitors (AT1-inhibitors), digoxin, alpha-1-receptor inhibitors) were the second most prescribed drugs (15.6 %) followed by antidepressants (9.9 %), anticonvulsives (6.1 %), proton-pump inhibitors (6.1 %), thyroxine (2.5 %), supplements (2.1 %), benzodiazepines (1.9 %) and antidiabetics (1.9 %).

### CDSSs - time expenditure

Thirteen patients had just a single prescription. Therefore, only 97 patient regimens remained for evaluation.

### CDSS Apotheken Umschau (AU)

93 pharmacotherapies were checked for DDIs with the CDSS AU. Due to the limitation of ten possible entered drugs, three patient’s regimens could not be evaluated. One regimen could not be evaluated because the CDSS neither recognized the drug name “Avamigran” nor its active agents. Evaluating the 93 pharmacotherapies required a total of 3:55:45 h. The maximum time for analyzing one pharmacotherapy was 6:22 min for a regimen comprising ten drugs. The minimum time was 1:02 min for a regimen comprising two drugs. The average time for analyzing one patient was 2:32 min.

### CDSS Medscape (MS)

Because the CDSS MS did not recognize various drugs or their active agents 52 pharmacotherapies could not be analyzed. Drugs not recognized by the CDSS MS were Phenprocoumon, Biperiden, Flupirtin, Tepilta suspension, Tolperison, Glibenclamid, Melperon, Metamizol, Lipoic acid, Macrogol, Opipramol, Tetrazepam, Zopiclone, Bromazepam. Moreover, drug combinations consisting of two drugs, e.g. Ramipril/HCT that had not been recognized as a combination but had been recognized when entered individually, were analyzed each on their own. The CDSS MS required a total of 1:28:35 h for evaluating the remaining 45 pharmacotherapies. 8:04 min was the maximum amount of time needed to analyze a patient’s regimen that covered 10 drugs. The minimum amount of time for an evaluation was 0:21 min for a regimen that comprised two drugs. The average time for analyzing one patient’s regimen was 1:58 min.

### CDSS AID Klinik (AID)

Ninety-seven patient pharmacotheapies were analyzed with the CDSS AID. All patients with two or more prescribed drugs fitted the systems criteria. All drugs were recognized and no patient regimen exceeded the limit of drugs that can be entered. Analyzing the 97 pharmacotherapies with the CDSS AID required a total of 3:12:27 h. 7.39 min was the maximum amount of time needed to evaluate one patient’s regimen comprising 20 drugs. The minimum amount of time required to analyze one patient’s regimen comprising a pharmacotherapy consisting of two drugs was 0:43 min. The average time needed to analyze one patient’s regimen was 1:59 min.

Table 2 shows the time expenditure of all three CDSSs by comparison.

**Table 2:** CDSS drug-interaction check – time expenditure

	CDSS AU	CDSS AID	CDSS Medscape
Regimens analyzed	93	97	45
Average time per analyzed regimen	2:32 min	1:59 min	1:58 min
Range	Minimum: 1:02 min	Minimum: 0:43 min	Minimum: 0:21 min
	Maximum: 6:22 min	Maximum: 7:39 min	Maximum: 8:04 min
Total time	3:55:45 h	3:12:27 h	1:28:35 h

*CDSS* Cinical decision suport sytem, *AID* CDSS AiD*Klinik*®, *AU* CDSS Apotheken Umschau, *MS* CDSS Medscape

### CDSS related time expenditure

Employing the CDSS AID was significantly faster than the use of CDSS AU (*p* < 0.05).

### Most frequently involved drug combinations and drugs

#### CDSS AU

When evaluating 93 pharmacotherapies comprising 446 drugs the CDSS AU discovered 261 DDIs in 63 (67.7 %) pharmacotherapies. In 30 (32.2 %) patient regimens no DDI was found by the CDSS AU. The maximum number of DDIs discovered was 12 in a regimen comprising ten drugs. The minimum number was one DDI discovered in a regimen comprising two drugs. The average number of interactions was 4.14 per patient. 3.8 % were classified as severe interactions, 36.3 % were severe or considerable interactions, 37.9 % of the discovered interactions were classified as only significant in some rare cases and 16.4 % were of slight relevance. 5.3 % of the discovered interactions were classified as most probably not relevant. The most frequently involved drugs were non-opioids (22 %), antidepressants (14.5 %) and opioids (10.1 %).

#### CDSS Medscape

The CDSS MS discovered 178 DDIs in 38 (84.4 %) pharmacotherapies comprising 202 drugs within a range of 1 to 17. The average amount of DDIs was 4.71. Of the 178 DDIs 6.7 % were classified as severe, 75.2 % as significant and 17.9 % as minor significant. Concerning all DDIs discovered by the CDSS MS, non-opioid analgesics (44.3 %) were the drugs involved most frequently, followed by beta blockers (7.8 %) and antidepressants (7.5 %). Only in 7 (15.5 %) of the 45 evaluated patient regimen no DDI was found.

#### CDSS AID

In analyzing 97 pharmacotherapies comprising 492 drugs, the CDSS AID discovered 170 DDIs within a range of one to twelve DDIs in 57 (58.7 %) pharmacotherapies. No DDI’s were found in 40 (41.2 %) of the evaluated patient regimens. The average number of interactions was 2.98 per patient. 11.7 % were clinically severe interactions, 42.9 % of the DDIs were classified as potentially clinically relevant interactions, and 45.2 % the discovered DDIs were clinically not relevant and not further specified. The most common drugs involved in DDIs according to the CDSS AID were non-opioids (27.9 %), ACE-inhibitors (12.9 %) and diuretics (11.2 %). Table 3 lists the drugs and drug combinations which most frequently contribute to DDIs.

**Table 3:** Drugs and drug combinations most frequently involved in DDIs

Detecting CDSS (interactions in total)		Drug combinations	%	Drugs	%
AU (261)	Severe (3.8 %)	ASS-Ibuprofen	80	ASS	40
		Melperone-Ropinirole	10	Ibuprofen	40
		Levofloxacin-Amitriptyline	10	Levofloxacin	5
				Amitriptyline	5
				Ropinirole	5
				Melperone	5
	Severe/Considerable (36.3 %)	Tramadol-Pregabalin	3.1	Pregablin	8.4
		Amitriptyline-Tramadol	3.1	Ibuprofen	7.8
		Ibuprofen-Enalapril	3.1	Amitriptyline	7.3
		Pregabalin-Morphine	3.1		
	Significant (rare) (37.9 %)	Ramipril-HCT	3	ASS	9.6
		Pantoprazole-ASS	3	HCT	8.5
		ASS-Olmesartan	3	Ibuprofen	6
		Metformin-ASS	3		
	Slight (16.4 %)	ASS-Amlodipine	14.9	ASS	32.5
		ASS-Ramipril	11.6	Amlodipine	8.1
		ASS-Bisoprolol	9.3	Thyroxine	8.1
		ASS-Metoprolol	9.3		
	Not relevant (5.3 %)	Pantoprazole-Diclofenac	21.4	Pantoprazole	21.4
		Pantoprazole-Duloxetine	21.4	Diclofenac	10.7
				Carbamazepine	10.7
				Duloxetine	10.7
MS (178)	Severe (6.7 %)	ASS-Ibuprofen	50	ASS	25
		Telmisartan-Ramipril	16.6	Ibuprofen	25
	Significant (75.2 %)	ASS-Ibuprofen	8.2	ASS	17.5
		Ibuprofen-Diclofenac	7.5	Ibuprofen	16.8
	Minor Significant (17.9 %)	ASS-Ibuprofen	18.7	Ibuprofen	21.8
		Diclofenac-Ibuprofen	15.6	ASS	18.7
AID (170)	Severe (11.7 %)	Ibuprofen-ASS	40	Ibuprofen	25
		Amitriptyline-Tramadol	15	ASS	20
		Trimipramine-Tramadol	10	Tramadol	12.5
	Potentially clinically relevant (42.9 %)	Ramipril-ASS	6.8	ASS	11.6
		HCT-Ramipril	4.1	Ramipril	8.9
		Enalapril-Ibuprofen	4.1	HCT	8.2
		Pregabalin-Oxycodone	4.1		

*CDSS* Cinical decision suport sytem, *AID* CDSS AiD*Klinik*®, *AU* CDSS Apotheken Umschau, *MS* CDSS Medscape, *HCT* Hydrochlorothiazide, *ASS* Acetylsalicylic acid

### Pain physician questionnaire

All five pain physicians believe that CDSS are important tools to prevent DDIs and improve medication safety. Four stated to use CDSSs on a regular basis. One physician stated to not use computerized check systems because of the increased workload and lack of time in hospital workflow. Two physicians have used all three check systems in the past. Two have only used CDSS AID and one physician has not used either of them.

### CDSS AU

Main advantages stated by the pain physicians were that AU is freely available, is easy to handle and has s self- explanatory layout. Disadvantages of the AU CDSS are that certain drugs are not recognized and too many irrelevant drug-drug interactions are highlighted. The explanations of potential DDIs are relatively long and written for laymen. Furthermore, no medication plans can be created and saved. Therefore, the drug regimens have to be reentered each time an interaction check is required. Additionally, minimal errors in drug spelling are not tolerated.

### CDSS Medscape

According to the pain physicians, advantages of the CDSS Medscape are its free availability, quick drug input, reasonable rating and concise explanation of interactions found. The main disadvantage was stated to be the fact that Medscape was created for English-speaking countries and drugs approved in USA only. This makes drug input for German drug names difficult and even impossible for drugs that are approved in Germany but not in the USA. Furthermore, no medication plan can be created and saved, forcing the physician to reenter all drugs every time changes are made to a patient’s drug regimen and when a DDI check is required. Minor errors in drug name spelling are not tolerated.

### CDSS AID

Main advantages were stated to be the easy handling and rapid drug entry with tolerance for spelling errors of drug names. A large data base helps to select all entered drugs. Ratings of the DDIs found are reasonable and explanations of the potential interactions are concise. Additionally, links to further sources of information about the drugs entered are provided. Drug plans can be created and saved. Patients regimens are clearly represented in tables.

According to the pain physicians, the main disadvantages of the CDSS AID are that it is available only at institutions which purchased the CDSS, and that the data entry is time consuming. For example, each drug has to be entered with dose and instructions for intake.

## Discussion

The immediate aim of this study was to determine the time needed to analyze pharmacotherapies of chronic pain patients for DDIs, using three different CDSSs. On a wider scale, the results of this trial serve to evaluate the usability of such tools in a daily routine.

We found that analyzing patient regimens employing the CDSS AU required on average 2:32 min detecting 4.14 interactions. The CDSS MS required 1:58 min for analyzing a pharmacotherapy detecting 4.71 DDIs in average. Using the CDSS AID to analyze pharmacotherapies required 1.59 min detecting 2.98 interactions on average.

The time needed to evaluate a patient’s pharmacotherapy, the DDIs found, as well as the drugs involved in the DDIs all depended on the particular CDSS used. For example, data entry differs among the CDSSs. While drugs are entered with dose and trade or generic name in the CDSSs AU and AID, no dose is required in MS, which can be time saving during drug entry. However, the fact that many German trade names were not recognized by the CDSS MS had an adverse effect on time expenditure. Similarly, the fact that the user had to look up the active agent before entering a patient’s regimen for drugs that were not known to the CDSS increased time expenditure and effort. Furthermore, even minor errors in the spelling of a drug name or active agent made it impossible for the CDSS MS and CDSS AU search engine to find the correct drug. This makes data entry difficult, especially when the real drug name is unknown. On the other hand, the CDSS AU automatically offers the option to enter additional information about patient diseases which requires extra time even if just declining the request. Moreover, the CDSS AU has not implemented a print option. This definitely prolonged the documentation because the desired information had to be extracted and printed using another program.

The way the results of the DDI check were provided by the three CDSSs differed as well. Both, the CDSS MS and AID delivered assorted information with more severe interactions listed first. In contrast, the CDSS AU did not sort the discovered interactions by degree of severity. This made evaluation of the results more difficult and the search for desired information more time consuming. Moreover, the number of the discovered DDIs as well as their classification varies between the check systems. The CDSS MS and AID each classify DDIs based on three degrees of severity, whereas the CDSS AU utilizes five. The number of DDIs discovered by the CDSS AU and the five categories of classification may lead to reduced awareness because too much information is provided [28, 29].

Concerning the usability of the CDSS MS there is an additional restriction because it was primarily designed for use in Anglo-American countries. Accordingly, in 53.6 % of the evaluated patient regimens, the CDSS MS did not recognize one or more drugs of the patient’s pharmacotherapies including some of the most frequently used analgesics in Germany. Even though the CDSS MS is a simple and easy to use tool for analyzing pharmacotherapies, it is of limited use for evaluating pharmacotherapies of chronic pain patients in German language area. In summary, when taking into account all different aspects, the CDSS AID turned out to be the fastest CDSS and proved superior in handling compared to the two other CDSS because it is especially tailored for a hospital workflow and to ease a physician’s work. These findings match the results of the implemented questionnaire and the qualitative comparison of the three CDSS by five pain physicians. All pain physicians chose the CDSS AID as preferred interaction check system. Especially the reasonable number of interactions found and the concise descriptions of DDIs make this CDSS popular among the physicians. The features that allow to create a medication plan that can be saved and that provide additional drug information make it easy to integrate this tool and the necessary interaction checks into a physicians work routine. Both other CDSSs turned out to be less practicable for different reasons. According to the pain physicians the CDSS AU seems to be a tool more suitable for patients than physicians. Too many of the discovered potential interactions are irrelevant and explanations of potential DDIs are too long. The freely available CDSS Medscape was found to be fast and sufficient with respect to identifying relevant interactions and to providing an adequate amount of additional information. Unfortunately, many drug regimens could not be evaluated for DDIs with this tool making it impossible for physicians to exclusively rely on this check system in a daily work routine.

The expenditure of time for analyzing patients’ pharmacotherapies is of great interest especially in view of the decreasing amount of time physicians have for patient treatment. According to a German study, general practitioners spend on average 8 min per patient assuming a number of 45 patients per day [30]. A two minute drug interaction check would extend the average patient treatment time by 25 %. However, this would apply only to patients who actually receive multiple drug prescriptions. Therefore, the overall increase in temporal effort and work-load for a physician is likely to be considerably smaller. Importantly, a modest increase in treatment time must be set in relation to the large numbers of DDIs that were actually detected in the patient’s pharmacotherapies and the obvious necessity to avert ADRs which in principal are preventable [6, 14, 18]. Keeping in mind that ADRs are a serious cause of morbidity, mortality and costs in healthcare [15–17] it seems more reasonable to spend a rather small amount of time for a DDI check than unnecessarily risking patient welfare or to invest a definitely greater amount of time and money in order to deal with the effects of ADRs. Indeed, just correcting a medication error in hindsight is more time consuming than an interaction check in advance [11].

### Limitations

The study setup reflects the performance of CDSSs in an actual hospital workflow. However, especially with respect to the check systems AID and AU only basic functions were applied. For example, aside from the prescribed drugs, no additional information was added to specify the interaction check. This could increase time expenditure in practice. In this regard, the CDSS AID provides an advantage as it is integrated into a computerized physician order entry system. Thus, additional patient information could easily be obtained and combined with the interaction check by the system increasing the accuracy of interaction checks [20]. This could lead to time savings compared to the internet-based CDSS where additional parameters and patient information have to be entered separately by the user. It must be mentioned, though, that the DDIs found by the different CDSSs were not examined for their correctness, their relevance, or their actual ADR risk. For example, the CDSS AU identified the drug combination Tramadol-Pregabalin as a severe risk for DDIs. Yet, the combination Tramadol-Pregabalin is part of current guidelines for pain therapy. This indicates that not every CDSS-based recommendation should be adopted without additional scrutiny which in turn can be time consuming. However, this example shows that mistakes in patient treatment can also occur from the use of CDSSs [26] and suggests that prior to implementation as treatment routine a CDSS should be carefully evaluated not only with respect to time expenditure but also for reliability.

In this study, patient regimens were entered sequentially in a single session by one person and a clear training effect was observed. This made data input significantly faster towards the final entries. However, increased time expenditure and other difficulties encountered especially during the process of getting acquainted with a new computer program could be of practical relevance. They could act as deterrents and provoke first time users to avoid the application of a CDSS. Moreover, the analysis of pharmacotherapies by the use of a CDSS was not only highly dependent on the examiner’s computer skills but also on his/her prior pharmacologic knowledge. For example knowing trade names and corresponding active agents ease both extracting information from patient records and entering the regimen into the CDSS.

From a technical point of view, the use of an internet-based CDSS may lead to differences in time expenditure due to location-/user-dependent differences in connection speed. Furthermore, the CDSSs were evaluated only for the expenditure of time arising from analyzing pharmacotherapies. The CDSSs were not evaluated concerning their sensitivity and the relevance of the discovered DDIs. However, in view of the observed discrepancies among the different CDSSs concerning the detected DDIs and the groups of drugs involved, this would be of further interest in future research.

## Conclusion

There are many reports that show that CDSSs undoubtedly are of relevance in preventing DDIs and their consequences. In our current study we determined the amount of time required for the use of a CDSS and compared their performance and practicability. Our results show that the commercially available CDSS AID is the preferred check system of pain physicians. Irrespectively of which CDSS is chosen, however, CDSS implementation would notably extend the time for patient treatment. In the authors’ opinion, the impact of this extra expenditure of time is outweighed by the long-term patient benefits including reduced ADR risks, morbidity and mortality. Therefore the authors recommend using CDSSs on a routine basis in clinical drug management during chronic pain therapy.
